# Mental Fatigue and Basketball Performance: A Systematic Review

**DOI:** 10.3389/fpsyg.2021.819081

**Published:** 2022-01-10

**Authors:** Shudian Cao, Soh Kim Geok, Samsilah Roslan, He Sun, Soh Kim Lam, Shaowen Qian

**Affiliations:** ^1^Faculty of Educational Studies, Universiti Putra Malaysia, Serdang, Malaysia; ^2^Faculty of Medicine and Health Sciences, Universiti Putra Malaysia, Serdang, Malaysia; ^3^Department of Physical Education, Wuhan Sports University, Wuhan, China

**Keywords:** mental fatigue, sports, athletic performance, recovery, basketball

## Abstract

Mental fatigue (MF) is a psycho-biological state that impairs sports-related performances. Recently, it has been proved that MF can affect basketball performance. However, a systematic overview detailing the influences of MF on basketball performance is still lacking. This study aims to investigate the effects of MF on the physical, technical, tactical, and cognitive performance of basketball. We used the databases of PubMed, Web of Science, SPORTDiscus, Scopes, and CKNI for articles published up to 31 May 2021. The articles included in this study were projected to test whether MF influences basketball athlete performance. Only experimental design studies were selected, and the control condition was without MF. Finally, seven articles fit the inclusion criteria. The results imply that MF impairs the technical aspects of basketball (free throws, three-point shots, and total turnover) and the players' cognitive [take-the-first (TTF) heuristics and decision-making] performance, which results in athletes not using their techniques skillfully and being unable to make practical decisions during critical points in the game. In addition to that, the influences of MF on physical and tactical performance have not been studied. Further studies should look into comprehensive research on the influences of MF on basketball performance, especially on a player's physical and tactical performance.

**Systematic Review Registration:** [https://inplasy.com/] [INPLASY2021100017].

## Introduction

Mental fatigue (MF) is a psychobiological state caused by prolonged, demanding cognitive activity, which has implicated many aspects of daily life. This condition causes an acute feeling of tiredness and a decreased cognitive ability (Boksem and Tops, 2008; Marcora et al., 2009; Van Cutsem et al., 2017). Along with the investigation of MF on cognitive performance, an increasing number of researchers focus on the potential impact MF has on one's physical performance. The most recent research aimed at understanding the phenomenon of MF found that it has a negative impact on athletic performance (Van Cutsem et al., 2017; Pageaux and Lepers, 2018; Habay et al., 2021), including endurance (cycling, running, yo-yo), motor skills (accuracy, speed, time taken to finish), and decision-making (errors, slower response time). MF harms the sports-specific psychomotor performances (SSPP) of different sports, including soccer, badminton, table tennis, basketball, and cricket (Habay et al., 2021). On the other hand, maximal force production (countermovement jumps, maximal voluntary contractions) was not affected by MF (Pageaux and Lepers, 2018).

According to the multiple resource model (Wickens, 1980), MF attracts peoples' attention as the secondary task which may simultaneously compete with the limited resource and potentially interfere with hazard recognition and take-over performance (Naujoks et al., 2017). In the higher demand condition, peoples' self-regulation mainly exhibited in the main task (Wandtner et al., 2016) and the adjustment in the interaction with the secondary task (Lin et al., 2019). On a control level, people regulate the current secondary task processing by disengagement with the secondary task if necessary, in which self-regulation was mainly manifested by peoples' attention allocation between main task and secondary tasks (Lin et al., 2019). As for sports area, sometimes, it is hard for athletes to perform to the best of their capabilities in fierce competitions, especially when success or failure has essential meaning. They have a higher cognitive burden to bear in those situations, which affects their performance (Nieuwenhuys and Oudejans, 2012). Studies suggest that the pressure of competition can induce anxiety and interfere with one's attention span, thus impairing one's athletic performance (Oudejans et al., 2011; Englert and Bertrams, 2012). Therefore, self-regulation was exerted to regulate their anxiety and better control their focus (Wilson et al., 2009; Englert and Bertrams, 2012; Baumeister et al., 2016; Englert, 2016), which can facilitate the execution of desired behaviors and task-relevant actions, thus leading to athletes being closer to their goal or highest standard of performance (Baumeister et al., 2016; Englert, 2016). However, exerting self-control may result in higher chances of failure in future efforts, called ego depletion, or MF (Baumeister et al., 2016).

Concerning basketball, people usually evaluate it by physical, technical, tactical and cognitive performance (Klusemann et al., 2013; Scanlan et al., 2014; Conte et al., 2018). Physical demands, such as stand-walk, jog, run, sprint, shuffle and jumps, are basic ability for playing basketball (Klusemann et al., 2013). Technical and tactical performance, such as the percentage of shot, rebounds, ball reversals and post entries are key factor between winning and losing teams (Conte et al., 2018). Cognitive components, such as perceptual and decision-making elements, influences basketball players' performance (Scanlan et al., 2014). Numerous studies have researched the influences of physical fatigue on players' performances and found that passing accuracy, ball speed and shooting technique significantly decrease when players are under the influences of physical fatigue compared to their non-fatigued states (Erculj and Supej, 2009; Li et al., 2021). However, these consequences are considered restricted to physical fatigue, and only a few articles have paid attention to the psychological aspect of basketball performance. During basketball games, players operate in high-intensity environments, which force them to conduct psychological operations to meet competition needs (Sighinolfi, 2020). Players who play at higher levels tend to have higher commitments, challenges, and confidence levels (Zarić et al., 2021). Therefore, due to basketball's cognitive and psychological demands, their cognitive engagement may cause MF.

An increasing number of articles have focused on the influences of MF in a variety of sports areas, including systematic reviews (Van Cutsem et al., 2017; Pageaux and Lepers, 2018; Kunrath et al., 2020; Habay et al., 2021). However, a systematic review investigating the influences of MF in the sport of basketball is lacking. Hence, this systematic review aims to identify the influences of MF on the physical, technical, cognitive, and tactical performance of basketball athletes.

## Methods

This systematic review was done following the PRISMA (2009) guidelines (Kosa et al., 2018). This title has already been registered on International Platform of Registered Systematic Review and Meta-analysis Protocols, and the registration number is INPLASY2021100017. It involved three steps: (1) a search on existing literature (including the selection of search terms, databases, and inclusion criteria); (2) a screening based on title; (3) a screening based on the article's abstract. The search was conducted on the 18 May, 2021, and the following databases were used: PubMed, Web of Science, SPORTDiscus, Scopes, and China National Knowledge Infrastructure (CNKI). The search terms were (“mental fatigue” OR “cognitive fatigue” OR “mental effort” OR “cognitive effort” OR “mental exertion” OR “ego depletion” AND “basketball”). In each database, a search was conducted by title. In addition to that, the related reference lists in the included articles were screened. Finally, there is not the language limitation.

**Figure 1:**
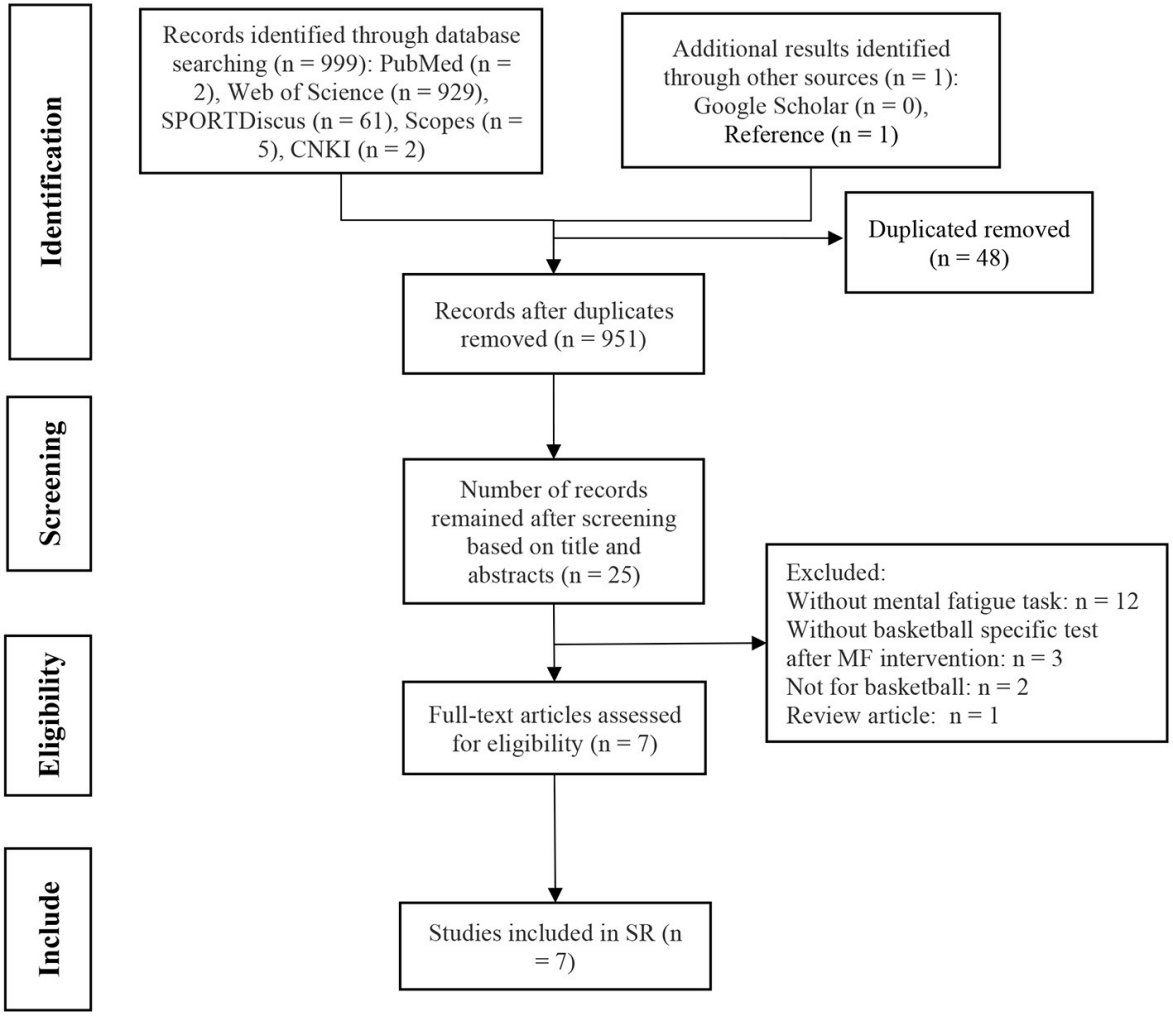
Systematic review search and screening procedure.

PICOS (population, intervention, comparison, outcome, study designs) criteria were used as the inclusion criteria, is presented in Table 1. Studies had to fulfill following inclusion criteria: (1) an evaluation of basketball-specific tests performed after the MF-inducing intervention was required, and, in the control group, the MF should not have been induced, or at least have triggered less MF than the intervention task; (2) the intervention was used to induce MF, and the sample population was comprised of basketball players; (3) measurements were implemented in a basketball-specific context (i.e., jump shots, dribbling, and passing); (4) the outcomes encompassed any form of basketball performance; and (5) randomized controlled trials (RCTs), non-randomized controlled trials (nRCTs), and non-randomized non-controlled trials (nRnCTs) had to be included. The data-collection process, based on PRISMA, is presented in Figure 1 (Tan et al., 2020).

**Table 1:** Inclusion criteria according to the PICOS conditions.

**Items**	**Detailed inclusion criteria**
Population	Basketball players (female/male) (without age restrictions)
Intervention	Mental fatigue
Comparison	Without mental fatigue
Outcome	Encompassed any form of basketball performance (physical, technical, cognitive and tactical)
Study designs	RCTs, nRCTs and nRnCTs

The results (titles and/or abstracts) of studies retrieved using the search strategy and the titles and/or abstracts of studies from other sources will be independently screened by two review authors to identify studies that may meet the above inclusion criteria. The reviewers will review these studies according to the standard of population, intervention, comparison, outcome, and study design. The two review authors will extract data independently, and the differences will be determined and resolved through discussion (discuss with the third author if necessary).

“QualSyst” was used to assess the methodology quality (Kmet and Lee, 2004). It contained 14 items (see Table 2). The score was set according the degree to which the certain criteria were met (yes = 2, partial = 1, no = 0). “NA” was marked when the items did not apply to the study design and excluded from the total calculation of score. A score of ≥75% indicated strong quality, a score of 55–75% indicated moderate quality, and a score of ≤ 55% indicated weak quality.

**Table 2:** Quality assessment “Qualsyst”.

**Items**	**Englert et al. (2015)**	**López et al. (2017)**	**Hepler and Kovacs (2017)**	**Moreira et al. (2018)**	**Bahrami et al. (2020)**	**Shaabani et al. (2020)**	**Filipas et al. (2021)**
I	2	2	2	2	2	2	2
II	2	2	2	2	2	2	2
III	2	1	1	2	2	2	2
IV	2	1	2	2	2	2	2
V	2	0	2	0	2	2	2
VI	0	0	0	0	0	0	0
VII	0	0	0	0	0	2	0
VIII	2	2	2	2	2	2	2
IX	2	1	2	2	1	2	1
X	2	1	2	2	2	2	2
XI	2	1	2	2	2	2	2
XII	0	0	0	0	0	0	0
XIII	2	2	2	2	2	2	2
XIV	2	1	2	2	2	2	2
Rating	Strong	Weak	Strong	Moderate	Strong	Strong	Strong

*NA, not applicable; 2, indicates yes; 1, indicates partial; 0, indicates no; I, question describe; II, appropriate study design; III, appropriate subject selection; IV, characteristics describe; V, random allocation; VI, researcher blinded; VII, subjects blinded; VIII, outcomes measure well defined and robust to bias; IX, sample size appropriate; X, analytic methods well described; XI, estimate of variance reported; XII, controlled for confounding; XIII, results reported in detail; XIV, conclusion supported by results*.

## Results

This study screened 999 articles, and 951 articles were remained after duplicates. After titles and abstracts were screened, 6 articles remained, and only 1 related article was found in screening the reference lists of the 6 articles (Figure 1). Overall, 7 articles were finally selected for the present systematical review. The result of quality assessment showed that 5 articles were of strong quality, 1 was moderate, and 1 was weak (Table 2). We did not exclude the article deemed to be of weak quality, because the number of articles in present systematic review was already limited.

### General Study Characteristics

Table 3 shows information on the study characteristics, which based on follow aspects: (1) Sample size: In totally, the seven articles had 258 subjects. The sample sizes ranged from 18 (Bahrami et al., 2020) to 72 (Shaabani et al., 2020) participants, and the mean sample size was 36.9 participants (SD = 23.4). (2) Gender: Most samples only contained males, but one sample (Hepler and Kovacs, 2017) had both genders. The percentage of female was 13.2% and male was 86.8%. (3) Population classification: Five articles involved trained athletes (Englert et al., 2015; López et al., 2017; Moreira et al., 2018; Bahrami et al., 2020; Shaabani et al., 2020), but other two included undergraduate students (Hepler and Kovacs, 2017) and amateurs (Filipas et al., 2021) respectively.

**Table 3:** Overview of the mental fatigue—inducing interventions.

**Study**	**Population**	**Characteristics**	**Intervention**	**Duration**	**Comparison**	**Methodological characteristics**	**Outcome**
Englert et al. (2015)	31 M	Professional; A = 29.26 ± 4.90	Transcribing a neutral text (omit all letters “e” and “n”)	6 min	Transcribing a neutral text without any instructions	RCT	Ego-depletion ↑ in I vs. C.
López et al. (2017)	18	Semi-professional; A = 21.35 ± 2.48	2 back-to-back memory tasks	Not reported	Oddball version	RCT, Crossover	MF ↑ in I vs. C.
Hepler and Kovacs (2017)	34 M; 34 FM	Undergraduate students; A = 20.66 years ±1.04;	Mental serial subtraction.	30 s	Counting backwards	RCT, Crossover	Not reported
Moreira et al. (2018)	32 M (U14: n = 14; U15: n = 10; U16: n = 8)	Trained; A = 15.2 ± 1.2 y; H = 180 ± 11 cm; Weight = 72 ± 15 kg; PHV = 2.1 ± 0.8 years	100% in-congruent modified Stroop color-word task	30 min	Easy cognitive task (10 min) + relaxing in room (20 min)	RCT, Crossover	Reaction time ↓ in time. Accuracy ↑ in time
Bahrami et al. (2020)	18 M	Trained; A = (22.2 ± 3.35)	Strop software Exercises +math tests.	120 min	Did not perform cognitive	nRCT	MF ↑ in I vs. C.
Shaabani et al. (2020)	72 M	Experienced; urban league; A = 28.6 ± 4.0; H = 193.0 ± 7.5 cm	Incongruent modified Stroop color-word task	15 min	Congruent modified Stroop color-word task	RCT, Crossover	Ego-depletion ↑ in I vs. C.
Filipas et al. (2021)	19 M	Amateur; A = 20 ± 3 years, H = 184 ± 6cm, Weight = 82 ± 6 kg	Watching tactical basketball video	30 min	Not reported	RCT, Crossover	Motivation = after I vs. C. MF ↑ in I vs. C.

*A, age; H, height; M, male; FM, female; PHV, peak height velocity; Y, year; C, control; I, intervention; RCT, randomized controlled trial; MF, mental fatigue*.

### Mental Fatigue-Inducing Interventions and Instruments

Two studies used a Stroop task to induce MF. The Stroop task was incongruent (Moreira et al., 2018; Shaabani et al., 2020) or combined with a Stroop software exercise involving math tests (Bahrami et al., 2020). Other studies used other forms of demanding cognitive tasks, such as watching a basketball tactical video (Filipas et al., 2021), N-Back tasks (López et al., 2017) and transcribing a neutral text with conditions (Englert et al., 2015). Hepler and Kovacs (2017) used mental serial subtraction to induce mental stress. Mental stress can induce anxiety and decrease attention. In those situations, self-control was exerted to regulate the pressure experienced and better focus their attention, which increases the possibility of self-control failure in the future, thus leading to ego depletion or MF (Shaabani et al., 2020). The duration of the intervention was different across studies, though most studies ranged from 6 to 120 mins (Englert et al., 2015; Moreira et al., 2018; Bahrami et al., 2020; Shaabani et al., 2020; Filipas et al., 2021). One article, however, only intervened for 30 s (Hepler and Kovacs, 2017), and another did not make mention of the duration (López et al., 2017).

The control conditions used by the included studies were varied. Englert et al. (2015) asked participants to transcribe the neutral text without any instructions. Moreira et al. (2018) used an easy cognitive task in which subjects sat in front of the computer screen for 10 mins and remained relaxed in the room for 20 mins. López et al. (2017) chose the oddball version of the control task. In this task, players had to press a button when a specific number was displayed onscreen. Hepler and Kovacs (2017) gave a two-digit number and asked participants to count backwards from it for 30 s in the control group. Shaabani et al. (2020) used a congruent-modified Stroop color-word task as the control condition. An overview of the MF-inducing interventions can be found in Table 3.

Several types of instruments were applied to test the effectiveness of reducing MF before and after cognitive tasks. First of all, five studies used one or more subjective manipulation checks: two studies used a visual analog scale (VAS) to assess MF and motivation (Bahrami et al., 2020; Filipas et al., 2021). Englert et al. (2015) applied a 4-item manipulation check to test whether the intervention induces the ego depletion. López et al. (2017) used the National Aeronautics and Space Administration-task load index (NASA-TLX) questionnaire to measure perceived effort and measure frustration workload score (0–100). Shaabani et al. (2020) used an ego-depletion manipulation check (EDMC) (four-item, 7-point Likert-type scale) to assess the depletion conditions between groups. All manipulation checks indicated an increase in MF in the experimental group. On the other hand, Moreira et al. (2018) used a behavioral manipulation check, in which reaction time and accuracy of the Stroop task were assessed. Finally, Hepler and Kovacs (2017) used a physiological manipulation check, in which a heart rate monitor (Polar H7—chest strap) was used to measure heart rate variability (HRV).

### Effects of MF on Basketball Performance

Basketball performance is divided into physical, technical, cognitive, and tactical performance for this section (Klusemann et al., 2013; Scanlan et al., 2014; Conte et al., 2018). Table 4 gives an overview of the mental fatigue on basketball performance.

**Table 4:** Overview of the mental fatigue on basketball performance.

**Study**	**Sample**	**Basketball task**	**Time**	**Outcome**
Englert et al. (2015)	31 M	30 FT	Post CT	FT accuracy ↓ I vs. C;
López et al. (2017)	18	30 FT	Post CT	Percentage of FT ↓ I vs. C
Hepler and Kovacs (2017)	34 M; 34 FM	Decision-making TTF	Post CT	TTF frequency, Number of options generated, first option quality and final decision quality = I vs. C; First option generation and final decision speed ↓ I vs. C
Moreira et al. (2018)	49 M	SSGs	Post CT	Efficiency = I vs. C; Total turnovers ↓ I vs. C
Bahrami et al. (2020)	18 M	3PS test	Pre and post CT	Percentage of 3PS ↓ I vs. C
Shaabani et al. (2020)	72 M	30 FT	Pre and post CT	Percentage of FT ↓ I vs. C.
Filipas et al. (2021)	19 M	60 FT	Post CT	FT accuracy ↓ I vs. C;

*3TS, three-point shot; FT, free throw; TTF, take the first heuristic; C, control; I, intervention; M, male; FM, female; CT, cognitive task; SSGs, small-sided-games*.

#### Physical Performance

The articles selected for the present study did not involve any MF on physical performance in basketball.

#### Technical Performance

Regarding technical performance, the six articles used the players' efficiency, total turnover in small-sided-games (SSGs), three-point shots, and free throws to evaluate a player's technical performance. Moreira et al. (2018) mentions an unclear difference in player efficiency between the two groups, but the total turnover increased in the intervention group, compared to the control group. Bahrami et al. (2020) says there was a significant decrease in the scores of the three-point shots from pre-test to post-test in the experimental group, but there was no noticeable difference in the control group. It also states that there was a significant difference in the scores of the three-point shots between the two groups involved in the post-test. As for the influences of MF on free throws, Englert et al. (2015), López et al. (2017), Shaabani et al. (2020), and Filipas et al. (2021) found that the percentage of free throws was lower in the experimental group than in the control group.

#### Cognitive Performance

Decision-making and take-the-first (TTF) heuristics were used to evaluate cognitive performance. Hepler and Kovacs (2017) found no significant difference in TTF frequency, the number of options generated, the first optional quality, or the final decision quality between two groups, but there was a noticeable difference between the first option generation and the final decision speed.

#### Tactical Performance

The articles selected in the present study did not involve the effects of MF on tactical performance in basketball.

## Discussion

In this study, we aim to sum the current extent of knowledge on the influences of MF on basketball players' performances. In order to achieve the aim, MF must first be successfully induced. Therefore, we reviewed the different methods that were used to attempt to cause MF. Overall, this review shows that MF can harm basketball players' performances in terms of efficiency, total turnover, free throws, take-the-first heuristics, decision-making, and three-point shots.

### Mental Fatigue-Inducing Interventions

In this study, the seven articles analyzed used six different tasks to induce MF. Whether the MF was induced successfully or not is very important.

Moreira et al. (2018) and Shaabani et al. (2020) used incongruent-modified Stroop color-word tasks of 30 mins to induce mental stress. Stroop tasks are a common way of inducing MF. For instance, Filipas et al. (2019) and Weerakkody et al. (2020) also used them in their studies of football and cycling (Filipas et al., 2019; Weerakkody et al., 2020). Rauch and Schmitt (2009) showed that a 15 mins Stroop task with 50% congruent and incongruent trials could induce MF. Englert et al. (2015) required participants to transcribe a neutral German text from the computer screen on paper for 6 mins as fast as they could. The participants in the experimental group had to omit all letters “e” and “n,” which are the most common letters in German. They had to override their writing habits, so that much self-control was needed. Another study also proved that the method was successful in inducing MF (Englert et al., 2015). Filipas et al. (2021) asked participants to watch a basketball tactical video to induce MF, and the results showed a difference between the experimental and control group. Video watching is relevant because it is expected of basketball players to perform video analysis conferences before competitions (Filipas et al., 2021). Therefore, making the experimental session similar to the actual basketball match should be considered in future studies. Bahrami et al. (2020) used the Stroop software and math tests to induce MF, but the duration was for 120 mins. It has been proposed that the different durations and difficulties of the mentally exerting tasks might have different influences on people (Van Cutsem et al., 2017). Hagger et al. (2016) states that sufficient duration and intensity are essential in inducing fatigue. López et al. (2017) used an N-Back test to induce MF, but the duration is not stated in the study. N-Back tests are tasks of continuous processing, and are also a standard method of inducing MF. Tanaka et al. (2009) proved that a 30 mins 2-Back test can cause MF (Tanaka et al., 2009). Hepler and Kovacs (2017) used mental serial subtraction to cause MF. In this task, participants needed to count backwards by seven from a 4-digit number, and they needed to complete as many correct answers a possible. If they gave a wrong answer, they had to start over from the first number. Previous studies proved that similar mental arithmetic could induce MF (Diller et al., 2011).

In summary, Stroop tasks, N-back tests, transcribing a neutral text with conditions and mental serial subtraction are not basketball-specific tasks. However, these tests require critical cognitive skills to achieve a high level of performance. Basketball belongs to the category of open skills, which requires players to react in unpredictable and changing, externally paced environments (Coyne et al., 2018). Basketball as a sport has some unique qualities. For instance, unlike soccer, the restrictions of the court and rules result in a high number of accelerations and decelerations, and more high-intensity displacements for the players (Halouani et al., 2014; Hoffmann et al., 2014). In addition to that, players also have to perform more offense-defense conversions in competitions, which means they need to frequently combine a series of skills (e.g., screen, fast break) and tactics (e.g., screen, fast break) in competitions (Pino-Ortega et al., 2021). Basketball may require better visual attention, action execution, and decision-making skills (Overney et al., 2008; Yarrow et al., 2009; Nakata et al., 2010), based on which MF can manifest subjectively, behaviorally, and physiologically. Nevertheless, little information about behaviors (e.g., reaction time and accuracy) were made mention to in cognitive tasks (Moreira et al., 2018). Hence, cognitive tasks should be considered in greater detail in future studies.

### Mental Fatigue and Basketball Performance

In order to discuss the subsequent basketball performance at a mentally fatigued state, a distinction was made between physical, technical, cognitive, and tactical performance.

#### Physical Performance

According to the cognitive load theory (Sweller, 1988), cognitive load refers to the used amount of working memory resources and heavy cognitive load has negative effects on task completion. Previous study reported that MF affected the information resources allocation of working memory, especially in the frontal and parietal regions which were closely related to working memory (Yang et al., 2021). Therefore, MF can increase the cognitive load leading to the reduction of performance.

In basketball competitions, physical performance includes stand-walks, jogging, running, sprinting, and low-, medium-, and high-intensity shuffles and jumps (Klusemann et al., 2013). The effects of MF on one's physical performance in basketball were not investigated. But Previous studies prove that MF impairs one's endurance, manifesting in increased completion time, decreased time before exhaustion, and self-selected power output/velocity (Van Cutsem et al., 2017). However, maximal strength, power, and anaerobic work are not affected by MF (Van Cutsem et al., 2017). Basketball combines aerobics and anaerobic exercises (Mancha-Triguero et al., 2020). Research shows that the average movement made by the world's best basketball centers is about 5,000 m per game, and the average movement of the world's best power forwards is about 6,000 m per game. An excellent attacking guard needs to move about 6,400 m to play a whole game. The moving distance of the China Basketball Association's (CBA's) leading players for a whole game range between 3,700 and 5,500 m, and the average moving distance per unit time per minute is 117–135 m per minute. There are significant differences among players in different positions, and the activity range of inside players is much smaller than those of outside players (Liu et al., 2012). Hence, whether the different positions the players play result in different degrees of MF should be investigated in the future.

Although the effects of MF on one's physical performance in basketball were not investigated in the chosen studies, other studies have proven its effects on the physical performance of other sports. For instance, Smith et al. (2015b) proved that the total distance covered on a treadmill, and shorter distances covered at lower speeds (Smith et al., 2015b), were decreased in mentally fatigued persons. Smith et al. (2015a) also verified that a yo-yo intermittent recovery test was performed at a decreased rate of 16.3% by mentally fatigued people (Smith et al., 2015a).

In conclusion, investigating whether MF impairs physical performance in basketball is necessary.

#### Technical Performance

In sports, technology refers to various processes, operation methods, and skills developed according to practical production experience and natural science principles. Basketball technology is a particular action done to achieve the goals of basketball, such as rebounds, assists, and scoring shots (Lan, 2001; Conte et al., 2018).

Six studies assessed the influences of MF on free throws, three-point shots, and total turnovers (Englert et al., 2015; Hepler and Kovacs, 2017; López et al., 2017; Moreira et al., 2018; Bahrami et al., 2020; Filipas et al., 2021). Those studies show the adverse influences of MF on technical basketball performance. The results show that the free throws and three-point shots in the experimental group were significantly lower than the control group, and Bahrami et al. (2020) added on to these results to state that there was no noticeable difference in the control group from pre-test to post-test. Comparatively, one article used small-sided-games (SSGs) to observe participants' technical performance (Moreira et al., 2018). SSGs are particularly relevant among the training methodologies used in basketball. SSGs can develop the physical, physiological, and technical-tactical aspects required in competitions (Klusemann et al., 2012; Delextrat and Martinez, 2013; Clemente, 2016). Moreira et al. (2018) recorded videos of its participants and used a formula to calculate the total turnovers of the two groups, thus proving that the total turnovers of participants in the experimental group were lower compared with control group.

From reviewing these articles, it was evident that none of the articles researched the effects of MF on technical performance in an official competition. To be specific, five articles researched the effects of MF on free throws and three-pointers during training (Englert et al., 2015; Hepler and Kovacs, 2017; López et al., 2017; Bahrami et al., 2020; Filipas et al., 2021), and one article researched it in SSGs (4 vs 4 in a court size of 28 × 15, in four sets of 2 mins and 30 s per set) (Moreira et al., 2018). Players have different mentalities and face different environments in training and in competition. Hence, the effects of MF on technical performance in official basketball competitions should be researched.

#### Cognitive Performance

Cognitive functions include a lot of basic mental operations, such as attention, memory, and executive functions involving working memory, decision-making, and multitasking (Lorenzo Calvo et al., 2021). Among these functions, attention is the one mainly defined as allocating cognitive resources to internal or external stimuli, which is key for sports performance (Furley and Wood, 2016).

Only one study assessed the effects of MF on TTF heuristics and decision-making outcomes (Shaabani et al., 2020). TTF heuristics are vital in sports (Johnson and Raab, 2003; Raab and Johnson, 2007). TTF refers to decisions made based on the first idea that springs to mind. As a result of the sequential order of option-generation, earlier options are better than options generated later in the process (Hepler and Kovacs, 2017). Hepler and Kovacs (2017) shows that MF does not affect the essential tenets of TTF. To be precise, in the study, participants were likely to choose their first choice in both experimental and control groups, and the number of options generated was identical. Therefore, MF did not affect the first option nor the subsequent options generated. As for decision outcomes, decision quality was not affected by MF, but the option-generation speed and final decision speed were significantly slower in the experimental group than in the control group. One study suggests that mental stress could inhibit reaction time (Van Gemmert and Galen, 1997).

Slimani et al. (2018) proved that MF had a negative effect on selection attention in concentration performances and increases the number of errors made (Slimani et al., 2018). When MF increased, brain activity gradually changed from negative to positive, which means that the inhibition of irrelevant information decreased the brain information system (Faber et al., 2012). Attention focus is a vital component of sports because there are many stimuli to which an athlete must attend. Attention can significantly impact performance when an athlete focuses (Milley and Ouellette, 2021). Therefore, researchers should research more into how MF influences cognitive performance in basketball.

#### Tactical Performance

Tactics refer to the “principles and methods of combat.” Basketball tactics are the principles and methods guiding individual skill and coordination among athletes in a basketball game. Usually, people divide tactics into offensive tactics (e.g., on-ball screen, off-ball screen) and defensive tactics (e.g., take the position, slide through) (Lan, 2001).

No articles have researched it in the present systematical review. However, a study proved that MF could impair tactical behaviors in soccer (Kunrath et al., 2020). Tactical performance has a crucial effect on basketball competitions. Winning teams have are more likely to have a higher number of ball reversals and post entries than losing teams (Conte et al., 2018), so investigating the influences of MF on tactical performance is vital.

To sum up the results, although the influences of MF on physical and tactical performance have not been researched, this systematic review shows that MF impairs technical and cognitive performance in basketball, which means that the percentage of shots will be decreased and the players' decision in competition will be affected when mentally fatigued. All these factors will likely lead to them losing the game.

Physical, technical, cognitive, and tactical performance are essential factors in basketball. Physical performance, such as running, sprinting, and shuffling, are the foundations on which a player operates their skills and tactics (McInnes et al., 1995), but the influences of MF on it in basketball have not been researched. On the other hand, players need to use unique techniques to execute strategies in basketball competition (Conte et al., 2015). In this regard, Basketball techniques, such as defensive rebounds and assists, are critical factors to win the game (Gomez et al., 2008). In this systematic review, although most of the reviewed articles researched the effects of MF on techniques, they did not do so during official competitions. Finally, cognitive performance, such as attention, anxiety, and motivation, can affect technical and tactical performance, which are also crucial in basketball competitions (Faber et al., 2012; Slimani et al., 2018; Milley and Ouellette, 2021), but only one of the six articles researched the influences of MF on cognitive performance.

In addition to studying the effects of MF on basketball performance, Moreira et al. (2018) also examined the effects of MF on salivary testosterone (T), cortisol (C), and alpha-amylase (sAA) responses. The increase of T and sAA concentrations were attenuated in the experimental group compared to the control group, but there was no change in C concentration between the two groups. The study suggests that the mentally fatigued state limits the increase of T and sAA. The T response might be relevant to the increase in errors during the small-sided-games (SSGs) (Boksem and Tops, 2008) and sAA is a biomarker for stress (Kivlighan and Granger, 2006; Silverman et al., 2010). C response is relevant to the effects of environmental and social-evaluative elements. In the cognitive task, or SSGs, these conditions were not present, which might be the reason that C concentration did not change between the two groups. On the other hand, Filipas et al. (2021) proved the negative effect a combination of MF and sleep deprivation has on technical performance for the first time (Filipas et al., 2021).

Finally, just one of the six articles mentioned a recovery strategy for MF (Shaabani et al., 2020). The study investigated mindfulness intervention effects (done for 15 mins) on mentally fatigued basketball players' free-throw performances. The participants in the control group listened to an audio-book segment on natural history, which was 15 mins long, and the results indicated that a brief mindfulness intervention could decrease the negative influence s of MF on basketball free-throws. Kabat-Zinn described mindfulness as “paying attention in a particular way, on purpose, in the present moment, and nonjudgmentally” (Sierpina, 2005). Mindfulness has been used in many sports to reduce mental stress. Reis Coimbra et al. (2021) proved that mindfulness interventions effectively attenuated MF in volleyball athletes.

Besides mindfulness training, there are other methods used for mental recovery in sports. For instance, Lorenzo Calvo et al. (2021) states that the intake of caffeine could improve attention (Lorenzo Calvo et al., 2021). Listening to self-selected music would be a suitable method of reducing MF in endurance performances (Lam and Phillips, 2019).

In conclusion, Shaabani et al. (2020) investigated mindfulness on free-throw tasks, but future studies should look at its effects on one's technical, physical, cognitive, and tactical performance (Shaabani et al., 2020). On the other hand, the recovery strategies used in basketball are limited, so further studies should also focus on finding more methods for basketball players to recover from MF.

## Limitations

Firstly, this review only included published articles. Therefore, the results might be affected by publication bias. Furthermore, two of the seven articles selected for the present study combined MF with sleep restriction and neuroendocrine respectively, which might have interfered with the results. Finally, the lack of articles on physical and tactical performance limits the overall understanding of knowledge on the effects of MF on basketball performance.

## Conclusion

By reviewing the results of seven published studies, the present study presents experimental evidence that most of the articles that investigated MF have concluded that it impairs technical performance (free throws, three-point shots) and cognitive performance (TTF heuristics and decision-making) in basketball. As a result of this, athletes may not perform to their utmost capability, which could lead to them losing the game. However, none of the articles investigated the effects of MF on physical and tactical performance in basketball. Hence, further studies should pay more attention to that. As the studies selected examined the influences of MF on basketball tests (e.g., free-throw tests and three-point shot tests), studies should be done on whether the influences of MF would be different in an actual competition. Finally, one article researched and proved a recovery strategy for impaired basketball performance, focusing only on free-throw performance. For the sake of helping athletes recover from MF, more methods should be investigated in the future.

## Data Availability Statement

The original contributions presented in the study are included in the article/supplementary material, further inquiries can be directed to the corresponding author/s.

## Author Contributions

The literature search and selection of studies were performed by SC and SG. Following an initial screen of titles and abstracts by SC, full scrutiny of potentially eligible studies was independently screened by SC and SG using the specific inclusion criteria. SR arbitrated any disagreements in study inclusion. Study quality assessment was performed by SC. All authors contributed to manuscript revision, read, and approved the submitted version.

## Conflict of Interest

The authors declare that the research was conducted in the absence of any commercial or financial relationships that could be construed as a potential conflict of interest.

## Publisher's Note

All claims expressed in this article are solely those of the authors and do not necessarily represent those of their affiliated organizations, or those of the publisher, the editors and the reviewers. Any product that may be evaluated in this article, or claim that may be made by its manufacturer, is not guaranteed or endorsed by the publisher.
